# Global Patterns of Geographic Range Sizes: A Bird's Eye View

**DOI:** 10.1371/journal.pbio.0040237

**Published:** 2006-06-20

**Authors:** Liza Gross

As earth's biodiversity continues to plummet, conservation biologists are redoubling their efforts to characterize the number and geographic range of the species that still inhabit it. But knowing how many species inhabit a given area (called species richness) and the extent of their range across the landscape is just one part of their strategy. Determining the mechanisms that underlie spatial variations in species' range size will help explain global patterns in species richness—why the tropics are biodiversity hotspots, for example—predict how global climate changes might affect biodiversity, and establish priorities for conservation.

In 1921, Frank Lutz, of the American Museum of Natural History, found that range area for North American plant species decreased “steadily and markedly” as one moved from high to low latitudes. Nearly 70 years later, this north-to-south decline in range size was codified as Rapoport's rule—but not without generating considerable debate about the universality of the principle. The question has remained controversial largely because it has been explored mostly at limited scales, with studies analyzing either small taxonomic groups or restricted regions within individual biogeographic realms (regions that roughly follow the divisions of the major continents).

In a new study, David Orme, Kevin Gaston, and colleagues revisit this issue by studying the global distribution of a major taxonomic group—birds—and show that spatial patterns of range size across the globe do not follow a simple north-to-south rule. The smallest range sizes are found on islands and mountain ranges, mostly in the southern hemisphere.

The researchers collated published data on breeding ranges for over 9,500 avian species, concentrating on sources that covered large geographical areas for a diverse set of species. (Their analysis excluded marine species because their ranges differ markedly from those of terrestrial species.) Breeding ranges were mapped as “vectors” (essentially shape fields) and imported into a grid that converts range area into appropriate cell sizes. Species range areas were calculated by totaling all the cells containing the species. Latitudinal extent was defined as the difference between the northern and southern limits of the vector maps for each breeding range. Species richness was calculated by adding all the species in each cell.

Their analysis shows that the majority of bird species have small geographic ranges. More than a quarter of species have ranges smaller than 86,872 square miles (equal to the area of Great Britain), with the smallest ranges found on islands, in low-altitude mountains, and throughout the southern hemisphere. The highest variation in range size is found in the northern hemisphere, particularly around the mid-latitudes.

Like the pattern for overall range size, the pattern for latitudinal range size was complex, with the smallest latitudinal extents found in mountainous regions and select island groups. In violation of Rapoport's rule, latitudinal range decreased from low to high latitudes in both hemispheres, rather than vice versa. Similarly, overall geographic range size did not decrease toward the tropics; although the largest ranges were at high northern latitudes, range size decreased toward the high southern latitudes. Even within individual biogeographic realms, range size increased with higher latitudes in only seven out of 13 cases.

There was a strong correlation between species richness and latitude, however, with the highest levels of biodiversity in the tropics, along with peaks in subtropical regions in the Andes, Himalayas, and the African Rift Valley. And there was a link, albeit weak, between species richness and range size, with high biodiversity areas harboring species with the smallest ranges.

With evidence that Rapoport's rule “does not generalize,” the researchers demonstrate the risks of drawing global conclusions about spatial variations in geographic range area based on limited biogeographical data. It takes a global view, they argue, to understand the true nature of these variations and the mechanisms that create them. For example, the finding that birds inhabit small ranges not only just in islands, which is not surprising, but also in tropical and subtropical mountain ranges suggests that it's not just the availability of land area that dictates range size but the availability of land area that exists within a climate zone that meets the species' adaptive needs. Future studies can test how broadly these spatial patterns occur in other taxa—essential information for understanding, and protecting, the current distribution of life on earth.

## 

**Figure:**
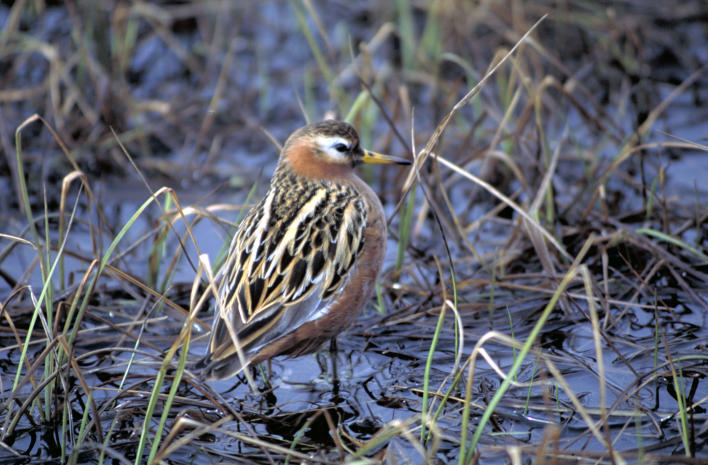
A global analysis of variation in the range sizes of birds shows that range area does not follow a simple latitudinal pattern. (Photograph: Red Phalarope; US Fish and Wildlife Service)

